# *EGFR* Mutations in Indian Lung Cancer Patients: Clinical Correlation and Outcome to EGFR Targeted Therapy

**DOI:** 10.1371/journal.pone.0061561

**Published:** 2013-04-19

**Authors:** Vanita Noronha, Kumar Prabhash, Abhishek Thavamani, Anuradha Chougule, Nilendu Purandare, Amit Joshi, Rashmi Sharma, Saral Desai, Nirmala Jambekar, Amit Dutt, Rita Mulherkar

**Affiliations:** 1 Department of Medical Oncology, Tata Memorial Hospital, Tata Memorial Center, Mumbai, India; 2 Department of Radiology, Tata Memorial Hospital, Tata Memorial Center, Mumbai, India; 3 Department of Pathology, Tata Memorial Hospital, Tata Memorial Center, Mumbai, India; 4 Advanced Centre for Treatment, Research and Education in Cancer, Tata Memorial Center, Navi Mumbai, India; Univesity of Texas Southwestern Medical Center at Dallas, United States of America

## Abstract

Screening for *EGFR* mutation is a key molecular test for management of lung cancer patients. Outcome of patients with mutation receiving EGFR tyrosine kinase inhibitor is known to be better across different ethnic populations. However, frequency of *EGFR* mutations and the clinical response in most other ethnic populations, including India, remains to be explored. We conducted a retrospective analysis of Indian lung cancer patients who were managed with oral tyrosine kinase inhibitors. Majority of the patients in the study had adenocarcinoma and were non-smokers. 39/111 patients tested positive for *EGFR* kinase domain mutations determined by Taqman based real time PCR. The overall response to oral TKI therapy was 30%. Patients with an activating mutation of *EGFR* had a response rate of 74%, while the response rate in patients with wild type *EGFR* was 5%, which was a statistically significant difference. Progression free survival of patients with *EGFR* mutations was 10 months compared to 2 months for *EGFR* mutation negative patients. Overall survival was 19 months for *EGFR* mutation patients and 13 months for mutation negative patients. This study emphasizes *EGFR* mutation as an important predictive marker for response to oral tyrosine kinase inhibitors in the Indian population.

## Introduction

The immense scientific advances made in the past decade have facilitated the in depth characterization of different disease subtypes, based on their genetic profiles. This has profound implications in non small cell lung cancer (NSCLC) which is the commonest cause of cancer deaths worldwide [1]. The treatment for NSCLC in the past was based mainly on patient related factors like the age, performance status and co morbidities. However, recent molecular advances have changed the treatment landscape of NSCLC. Key molecular changes like mutation in the epidermal growth factor receptor (*EGFR*) are involved in cell proliferation and cell survival in the neoplasms [2], [3].

It has been observed that patients who respond well to an EGFR inhibitor harbor certain mutations in the *EGFR* exons 18, 19 or 21. These mutations serve as markers for predicting the response in patients to oral tyrosine kinase inhibitors targeted to the EGFR tyrosine kinase. An additional mutation in *EGFR* exon 20 is known to be responsible for acquired resistance to this therapy [4]. EFGR tyrosine kinase inhibitors (TKI) have revolutionized the therapy of NSCLC. In patients whose tumors harbor the *EGFR* mutation, the use of an EGFR TKI has led to increased response rate and prolongation of progression free survival [5]. *EGFR* mutations are more likely to occur in patients of Asian origin, who are female, never-smokers and have adenocarcinoma [6]. However, there is very little information regarding occurrence of *EGFR* mutations in the Indian population and the activity of EGFR TKI. There is only one study reported from India on *EGFR* mutations in lung cancer, which focuses mainly on the epidemiology of patients who harbor these mutations [7].

We present the first study from India which correlates the EGFR mutation status of patients, with their clinical outcome when treated with oral EGFR TKI. Our study was aimed at carrying out mutation detection in the DNA extracted from Formalin Fixed Paraffin Embedded (FFPE) lung biopsies of NSCLC patients, and to correlate the mutation status with the response and the the clinical outcome of the patient to EGFR targeted therapy.

## Materials and Methods

The present study was a retrospective analysis of patients with advanced NSCLC receiving oral EGFR TKI, in whom the EGFR mutation status was determined. The project was approved by the Institutional Review Board (IRB) and the Ethics Committee (EC) of Tata Memorial Hospital (Mumbai, India). This study was monitored by data monitoring committee of Tata Memorial Hospital. Since this was a retrospective analysis, the IRB and the EC waived the need for an informed consent. Patients were randomly selected based on the availability of biopsy block from the database maintained in the Medical Oncology Department at Tata Memorial Hospital. These patients were started on oral TKI as part of standard care. DNA extracted from FFPE blocks was analyzed for EGFR mutation status. The result of the mutation status was blinded to the treating Physician. Information collected included demographics, baseline characteristics including smoking status, histopathology and clinical outcome including toxicity assessment, response to TKI, progression, therapy at progression and survival. Response was evaluated according to RECIST v 1.1. Toxicity was graded according to CTCAE, v4.03. Progression was defined as clinical deterioration or radiological progression. CT scans were done every 2 to 4 months or depending on patient's symptoms. Data was analyzed using SPSS, v 15. Progression-free survival was calculated from the date of starting oral TKI to the date of progression (either symptom deterioration or radiologic progression), or death from any cause. Overall survival was calculated from the date of diagnosis to death from any cause. Median follow-up was calculated for the surviving patients from date of diagnosis to the date of last follow-up. The study was conducted in accordance with the declaration of Helsinki and the International Conference on Harmonization Guidelines for Good Clinical Practice.

### Collection of patient samples

The FFPE blocks of the patients were collected from the Pathology department of Tata Memorial Hospital. The hematoxylin and eosin stained sections from the blocks were mounted on slides and viewed under the microscope and it was confirmed that the tumor – region constituted more than 75% of the tissue mass.

### Mutation analysis by TaqMan based real time PCR technique

Taking into account the high frequency of occurrence of specific mutations in different populations around the world, it was decided to carry out TaqMan based real time PCR technique for mutation detection with the help of probes that can anneal specifically to the mutant or wild type allele. The mutations studied were in frame deletions in exon 19, L858R point mutation in exon 21, and the G719C point mutation in exon 18. The assay was carried out in 384-well reaction plates (Applied Biosystems), and the reaction was carried out in 5 µL, which contained 2.5 µL of the Taqman mastermix (Applied Biosystems), primers at a final concentration of 9 µM and probes at a final concentration of 2 µM; the remaining volume was made up to 5 µL with PCR grade water. The reaction was carried out at 50°C for 2 minutes and 95°C for 10 minutes, followed by 40 cycles of 95°C for 15 seconds and 60°C for 1 minute, in the Applied Biosystems 7900 HT machine.

### Statistical tests

All statistical analyses were carried out using the SPSS software version 15.0. The difference between proportions was compared by Chi square test and the significance value was set at 0.05. Kaplan Meier curve was plotted for the progression free survival and the overall survival in months. Log rank test was used to compare the PFS and OS in different groups.

## Results

### Characteristics of the study population

Between January 2010 and July 2012, there were 111 patients who were enrolled in the study from whom a biopsy sample was available, mutation detection was successfully performed, oral TKI was used as therapy and full clinical details were available. The demographics of the patients are shown in the table (Table 1).

**Table 1:** Patient demographics, clinical characteristics and details of EGFR mutation.

**Total number of patients**		111
**Median age (yrs)**		55
**Gender**	Males	58
	Females	53
**Smoking status**	Smokers	21
	Non-smokers	88
**EGFR mutation status**	Mutation positive	39
	Mutation negative	72
**Type of EGFR mutation**	Exon 19 In-frame deletions	29
	Exon 21 L858R mutation	9
	Exon 18 G719C mutation	1
**Histopathology**	Adenocarcinoma	107
	Squamous cell carcinoma	4
**Performance status**	ECOG 0	11
	ECOG 1	39
	ECOG 2	40
	ECOG 3	14
	ECOG 4	4
**Site of metastasis**	Pleural effusion	32
	Brain	11
	Lung	36
	Bone	27
	Other sites	25
**Line of therapy in which oral TKI was used**	First line	92
	Second line and beyond	19

### TaqMan based real time PCR based screening for *EGFR* mutations

Mutation detection results were positive in 39 patients. Among these, 29 patients were detected to be positive for the in frame deletion in exon 19. The L858R point mutation in exon 21 was observed in 9 patients and the G719C point mutation in exon 18 was observed in 1 patient. Most of the above mutations were heterozygous, except in one patient where the L858R mutation was found to be a homozygous variant (Table 1).

### Clinical Correlation and response to oral tyrosine kinase inhibitor (TKI)

Among the 39 patients, who were found harboring the activating mutations, 29 patients had a partial response to oral TKI therapy, 6 patients had stable disease, while 4 patients had progressive disease as the best response (Table 2). In the 72 patients in whom no activating mutation was observed, 4 patients had a partial response, 22 patients had stable disease and 46 had progressive disease. Thus the response rate to oral TKI for mutation positive patients was 74%, while the response rate in mutation negative patients was 5%. The Chi-square test revealed a significant correlation between the mutation status of the patient and the response observed, with a p value<0.001.

**Table 2:** EGFR mutation status of the patients with clinical correlation.

	EGFR mutation negative (n = 72)	EGFR mutation positive (n = 39)	Statistical test
**Smoking Status**			
Smokers	17	4	Pearson Chi-square test: p:0.075
Non-smokers	53	35	
**Gender**			
Males	46	12	Pearson Chi-square test: 0.001
Females	26	27	
**Histopathology**			
Adenocarcinoma	69	38	Pearson chi square test: p = 0.67
Squamous cell carcinoma	3	1	
**Clinical Response**			
Partial Response	4	29	Pearson Chi-square test:0.000
Stable Disease	22	6	
Progressive Disease	46	4	
PFS (months)	2	10	Long rank test (Mantel-Cox): 0.000
OS (months)	9	19	Long rank test (Mantel-Cox): 0.001

### Survival by *EGFR* mutation status

**Figure 1:**
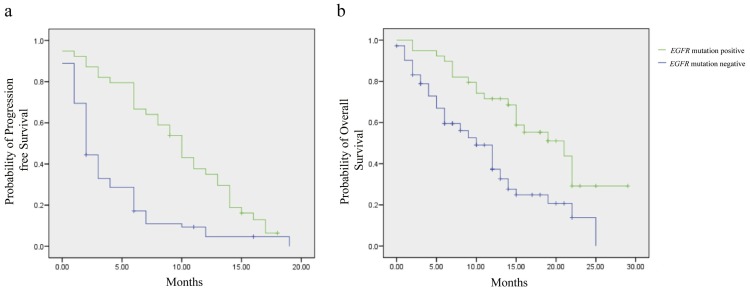
Survival by *EGFR* mutation status. (A) Progression-free survival (PFS) for the *EGFR* mutant patients was 10 months (95% CI: 8–11.9 months), while the estimated median PFS for *EGFR* mutation negative patients was 2 months (95% CI: 1.5–2.5 months), p = 0.000 by log rank test (Mantel Cox). (B) Overall survival (OS) for *EGFR* mutant patients was 21 months (95% CI: 12.4–25.6 months), while the estimated median OS for *EGFR* mutantion negative patients was 10 months (95% CI: 7.4–12.6 months), p = 0.001 by log rank test (Mantel Cox).

The median follow-up was 18 months (range: 16.4 to 19.7 months). The estimated median PFS for the entire cohort of patients was 4 months (95% CI: 2.5–5.5 months). The estimated median PFS for the EGFR mutant patients was significantly longer at 10 months (95% CI: 8–11.9 months) as compared to the estimated median PFS for EGFR negative patients which was 2 months (95% CI: 1.5–2.5 months), p = 0.000 by log rank test (Mantel Cox) (Figure 1a). The estimated median OS for all patients was 13 months (95% CI: 10.7–15.3 months). The estimated median OS for EGFR positive patients was 21 months (95% CI: 12.4–25.6 months), while that for EGFR negative patients was 10 months (95% CI: 7.4–12.6 months), p = 0.001 by log rank test (Mantel Cox) (Figure 1b).

### Toxicity associated with oral tyrosine kinase inhibitor (TKI)

The main toxicities noted were skin and gastrointestinal, as detailed in Table 3. Other toxicities noted in 2 patients included anorexia, fatigue and mucositis. 62 patients experienced no toxicity.

**Table 3:** Toxicities.

	None	Grade 1	Grade 2	Grade 3
Skin toxicity	77	10	10	14
GI toxicity	103	5	2	1

## Discussion

Worldwide, it is now well known that molecular markers, especially EGFR activating mutations, identify a subset of patients with NSCLC whose outcome is better with tyrosine kinase EGFR targeted therapies [5]. However, there is a lack of data from India regarding EGFR mutation and the response and outcome of these patients when treated with tyrosine kinase EGFR inhibitors. We present the first clinical data from India regarding EGFR mutation in NSCLC patients and the clinical outcome of these patients to oral TKI therapy.

The majority (81%) of the patients in our study were non-smokers, a significant proportion (48%) were females and most of the patients (96%) had adenocarcinoma. Thus, clearly this was a clinically enriched population who were chosen for oral TKI therapy based on clinical parameters and our cohort may not be truly representative of the actual lung cancer patient pool in India [8], [9].

In our study, 39 out of 111 patients, i.e. 35% of the patients were found to harbor an *EGFR* mutation. The previous study from India found that the mutation rate was 51.8% [7]. It is likely that both our study and the prior Indian report overestimated the incidence of *EGFR* mutation, because of a small sample size and clinically selected patients. Worldwide, the incidence of *EGFR* mutations has been well characterized and has been reported to occur at the rate of 10–15% in North Americans and Europeans, 19% in African-Americans and about 30% in East Asians [10]–[13]. We found that 35 of the 39 patients with *EGFR* mutations (90%) were non-smokers, while 53 of the 70 patients who were EGFR mutation negative (76%) had a smoking history. Regarding a gender predilection, 27 of the 39 patients with *EGFR* mutations (69%) were female while 46 of 72 patients with *EGFR*-negative tumors (64%) were male. Given that the overwhelming majority of patients in our study had adenocarcinoma (96%) and all patients were from India, it is impossible to comment on the correlation of pathology or ethnicity to *EGFR* mutation status.

Regarding the type of *EGFR* mutations detected, 74% of the patients were noted to have in frame deletion in exon 19, 23% had the L858R point mutation in exon 21 and only 2.5% patients had the G719C point mutation in exon 18. In the reported literature, approximately 45 to 54% of EGFR mutations are in-frame deletions in exon 19, while approximately 40% of *EGFR* mutations are missense mutations in L858R in exon 21 and between 4 to 9% of the mutations were reported in exon 20 [5].

The toxicity noted in our patients was similar to that described in the literature, although less toxicities were noted than what have been previously described. We did not have any case of interstitial lung disease as a result of oral TKI therapy. In the IPASS study, 66% of patients developed rash and 47% of the patients experienced gastrointestinal toxicity. The retrospective nature of our analysis may be one of the reasons why toxicities were noted to a lesser extent; however Indian patients may experience less toxicities as a result of EGFR targeted therapies due to various factors like ethnicity, dark skin, different dietary patterns and other racial differences.

The overall response to oral TKI therapy was 30%. Patients with an activating mutation of *EGFR* had a response rate of 74%, while the response rate in patients with wild type EGFR was 5%, which was a statistically significant difference, p<0.001. This is very similar to what has been reported in the literature, with a response rate of 72% in mutant positive patients, and a response rate of 1.1% in mutant negative patients [5]. The slightly higher response rate in our mutant negative patients probably indicates that we were not able to detect the *EGFR* mutation, when it was in fact present, or that there were other genetic events in the *EGFR* or alternative pathway that conferred sensitivity to oral TKI, in spite of lack of *EFGR* mutation. Other studies report varying response rates to oral TKI in *EGFR* negative patients. In a study by Han et al, the response rate to Gefitinib was 25.9% in EGFR mutation negative patients, compared to 84.6% in EGFR mutant patients [14]. Yang et al reported a 20% response rate to Gefitinib in *EGFR* negative patients while Han et al reported a response rate of 13.7% [14], [15]. The reason for the wide range of response rates to oral TKI therapy in patients, who are not detected to carry an *EGFR* activating mutation, is due to the varying sensitivities of the method used to detect the *EGFR* mutation. In our study, 4 patients who were detected to harbor activating mutations in the EGFR tyrosine kinase domain were found to be resistant to Gefitinib at their 2 to 3-month follow-up scan. This might be possibly due to the development of secondary mutations resulting in an acquired resistance to EGFR targeted therapies. Thus it is necessary to look at more markers for the effective prediction of the response to EGFR-TKIs and it is also necessary to obtain biopsies of the primary tumor subsequently during the course of the treatment to detect the presence of secondary mutations that could alter the response of the patients to the drugs [6].

In terms of survival, the estimated progression free survival (PFS) for all the patients was 4 months. The PFS for patients with EGFR mutation was significantly longer at 10 months, as compared to an estimated PFS of 2 months for *EGFR* negative patients, p = 0.000 by log rank test. The estimated overall survival for all patients was 13 months. The estimated median OS of the patients with *EGFR* activating mutations was significantly longer at 21 months, as compared to an estimated median OS of 10 months for *EGFR* negative patients, p = 0.001 by log rank test. In the updated survival results of the IPASS study, the median PFS in mutation positive patients was 9.5 months versus 1.5 months for mutation negative patients, while the OS in mutation positive patients was 21.6 months versus 11.2 months in *EGFR*-negative patients. In their study on patients with *EGFR* activating mutations, Maemondo et al reported a PFS of 10.8 months and an OS of 30.5 months following gefitinib therapy [16]. Thus, the survival results in our patients are similar to the results previously reported in the literature.

Thus, Indian patients with EGFR activating mutations have a significantly better response rate, progression free survival and overall survival when treated with EGFR targeted therapies.
